# Novel approach of using a cocktail of designed bacteriophages against gut pathogenic *E. coli* for bacterial load biocontrol

**DOI:** 10.1186/s12941-014-0039-z

**Published:** 2014-07-26

**Authors:** Ahmed Sahib Abdulamir, Sabah AA Jassim, Fatimah Abu Bakar

**Affiliations:** 1 Microbiology Department, College of Medicine, Alnahrain University, Baghdad 14222, Iraq; 2 Applied Bio Research Inc., 455 Pelissier St., Ontario N9A 6Z9, Windsor, Canada; 3 Faculty of Food Science, University Putra Malaysia, Serdang 43400, Selangor, Malaysia

**Keywords:** Bacteriophage, Animal feed, Biocontrol, E. coli, Bioprocessing, Phage design, Phage cocktail

## Abstract

**Background:**

This study was conducted to explore new approaches of animal biocontrol via biological control feed.

**Method:**

White rats were subjected to 140 highly lytic designed phages specific against *E. coli*. Phages were fed via drinking water, oral injection, and vegetable capsules. Phage feeding was applied by 24 h feeding with 11d monitoring and 20d phage feeding and monitoring. Group of rats received external pathogenic *E. coli* and another group did not, namely groups A and B.

**Results:**

Phage feeding for 20d via vegetable capsules yielded the highest reduction of fecal *E. coli*, 3.02 and 4.62 log, in rats group A and B respectively. Second best, feeding for 20d via drinking water with alkali yielded 2.78 and 4.08 log in rats groups A and B respectively. The peak reduction in *E. coli* output was 5–10 d after phage feeding. Phage control declined after 10^th^ day of feeding.

**Conclusions:**

The use of cocktail of designed phages succeeded in suppressing flora or external *E. coli*. The phage feed biocontrol is efficient in controlling *E. coli* at the pre-harvest period, precisely at the 6^th^-8^th^ day of phage feeding when the lowest *E. coli* output found.

## Background

*Escherichia coli* (*E. coli*) is the predominant nonpathogenic facultative flora of the human and animal intestine [1]. However, several strains of *E. coli* have developed the ability to cause diseases in humans. Strains of *E. coli* that cause gastroenteritis in humans can be grouped into six categories: enteroaggregative (EAEC), enterohemorrhagic (EHEC), enteroinvasive (EIEC), enteropathogenic (EPEC), enterotoxigenic (ETEC), and diffuse adherent (DAEC) [1].

Among the most notorious, EHEC is a variety of *E. coli* that produces large quantities of one or more related potent toxins that cause severe damage to the lining of the intestine. These shiga-like toxins act on vero cells of colorectum [2]. It is transmitted to humans through contaminated food, water, and direct contact with infected people or animals [3].

The infectious dose of EHEC and other pathogenic *E. coli* is very low, under 100 organisms [4] which requires stringent measures to decrease *E. coli* load from animals and environment. The organism can be found on most cattle farms and it is commonly found in petting zoos and can live in the intestines of healthy cattle, deer, goats, and sheep [5]. Therefore, meat can become contaminated during and after slaughtering, and organisms can be accidentally mixed into meat when it is ground [6]. Moreover, *E. coli* is highly present on the cow’s udders or on equipment used in milking which may get into raw milk [7]. Therefore, drinking milk or eating meat, especially ground beef that has not been cooked sufficiently to kill *E. coli,* can cause infection given that contaminated meat looks and smells normal. This problem has not yet been worked out due to the lack of the appropriate measures to lower, or if possible, abolish the *E. coli* burden from animal gut before or during slaughtering. Therefore, novel approaches of animal biocontrol against *E. coli* might be necessary to pursue, including bacteriophage-based biocontrol.

Phage biocontrol represents the application of specific phages, which are pathogens of bacteria, to selectively reduce or eliminate pathogen-susceptible organisms from specific natural environments (e.g., the bodies of humans and other animals), artificial environments (e.g., farms, factories, offices, hospitals, etc.), or even laboratory environments (e.g., to reduce streptomycete numbers on soil dilution plates [8],[9]. The ability of phages to recognize precisely their hosts, rendered them as favorable antibacterial agents because broad-spectrum antibiotics kill target bacteria along with other beneficial bacteria present in the farm or in the organism body, say intestinal flora [10]. Many studies were applied mainly in the Soviet Union counties showed that application of phages in bacterial therapy or biocontrol is attainable in theory but practicably results were not so successful due to the lack of full coverage of target bacteria and the rapid emergence of bacterial mutations leading to complete resistance against phage infection [11]–[14]. Therefore, phage therapy or phage biocontrol have become unsuccessful [15] and eventually led to replacement of phage therapy with antibiotic treatment [12].

The exploitation of bacteriophages as a realistic approach to the control of pathogens has attracted considerable interest in recent years [10],[14] because of the emergence of antibiotic resistant bacteria. For example, calves and piglets with diarrhea due to experimentally administered pathogenic *E. coli* were cured within 8 h following phage administration [16]. Experimentally induced diarrhea could be prevented by spraying the litter in the calf rooms with aqueous phage suspensions or by keeping calves in uncleaned rooms previously occupied by calves whose *E. coli* infection had been treated by phage administration [17]. Hence, elimination of the pathogenic *E. coli* at the preharvest stage could play a significant role in preventing its introduction the food chain [18]. Bactericidal bacteriophages may provide a natural, nontoxic, feasible approach for controlling several human pathogens [11] since phages are part of both gastrointestinal and environmental ecosystems [19].

Unfortunately, the lack of techniques to counter phage sequestration, resistance, and conversion eventually led to replacement of phage therapy with antibiotic treatment [12]. However, with our exclusive knowledge of phage breeding techniques, it may be possible to circumvent the problems encountered in previous attempts to use phages as natural antimicrobial agents. In this study, a cocktail of 140 specific lytic phages, which were previously optimized and bred by non-genetic breeding techniques bacteria [IPO-UK Patent Application No. 0822068] was used in novel approaches in animal biocontrol and animal feed. White rats were used for testing the animal feed with anti-*E. coli* phage cocktail. The intestinal load of *E. coli* as well as experimentally inoculated human pathogenic *E. coli* were then traced by microbiological methods to estimate the phage-driven decline of the *E. coli* microbial load in the treated animals.

## Methods

### Preparation of the anti- *E. coli* phage cocktail

#### Media

Luria broth (LB): tryptone 10 g l-1 (HiMedia, Mumbai, India), yeast extract 5 g l-1 (HiMedia, Mumbai, India), and sodium chloride 10 g l-1 (HiMedia, Mumbai, India) at pH 7.2 were used in all the protocols. L-agar (LA), consisted of the above with the addition of 14 g l-1 agar (HiMedia, Mumbai, India) was used for culture maintenance. Bacterial dilutions from 18 h LB cultures grown at 37°C were carried out in phosphate buffered saline (PBS, Oxoid, UK). For plaque assay, the ‘soft layer agar’ used was LB prepared in Lambda-buffer [6 mmol l-1 Tris pH 7.2, 10 mmol l-1 Mg(SO4)2.7H2O, 50 μg ml-1 gelatin (Oxoid, UK)], was supplemented with 4 g l-1 agar bacteriology No. 1 (HiMedia, Mumbai, India).

#### Bacterial strains

Four hundred and thirty clinical isolates of EHEC and non-EHEC *E. coli* were obtained from hospital inpatients (Microbiology laboratories, Hospital Serdang and Hospital Kajang in Selangor, Malaysia) including documented sporadic cases of haemorrhagic colitis, non-haemorrhagic colitis, urinary tract infections, infected wounds, vaginitis, and bacteremic cases. They were reconfirmed by using Microbact GNB 12A system (Oxoid, UK), a microtitre well-scaled chemical test. Microbact system has 100% sensitivity for identifying *E. coli* from other Enterobacteracea bacteria.

In addition, several *E. coli* reference strains were used: one EHEC NTCC 129001 and five non-EHEC (two are generic strains; ATCC 12799 and NTCC 9001, three human enteropathogenic strains (EPEC); ATCC 12810, ATCC 25922, and ATCC 35218 (zoonotic). Both *E. coli* clinical isolates and representative NTCC and ATCC *E. coli* strains were used throughout phage isolation, propagation, optimization and breeding as described here. The strains were maintained on L-agar plates and transferred bimonthly. All cultures were stored at −20°C in 15% glycerol. Prior to investigation a stock culture of the bacteria was maintained on LA plate. One loopful of the bacterial strain was inoculated into a 100 ml Erlenmeyer flask containing 10 ml of LB and incubated for 18 h at 37°C and 90 rev min-1 in an incubator shaker (Innova 4000, New Brunswick Scientific). For experimental tests appropriate serial dilutions were made in LB.

#### Bacteriophages

Wild bacteriophages (phage) used in this study were isolated from and specifically designed for 430 clinical isolates and 6 reference strains of EHEC (http://www.sumobrain.com/patents/wipo/Methods-bacteriophage-design/WO2010064044A1.pdf). The phage master mix was composed of 140 phages that were previously isolated, bred, and produced by 2 types of novel, under patenting breeding techniques (http://patentscope.wipo.int/search/en/WO1995023848): chemical vertical breeding which is characterized by enhancing the lytic infective criteria of the bred phages in order to obtain optimized biokinetic potential and chemical horizontal breeding which is characterized by altering the specificity of the bred phages to be reoriented to new strains of *E. coli* leading to wider coverage of target bacteria (http://www.sumobrain.com/patents/wipo/Methods-bacteriophage-design/WO2010064044A1.pdf). This breakthrough technology opened doors for designing and optimizing unprecedented phage applications including eliminating the pathogenic EHEC and non-EHEC bacteria from food, machinery tools, and medical instruments by using high number of artificially bred specific phages. The resultant phages were mixed together forming what is called the ‘phage master mix’. The phage master mix was composed of 140 highly lytic and specific bred phages.

#### Groups of tested animals

**Figure 1:**
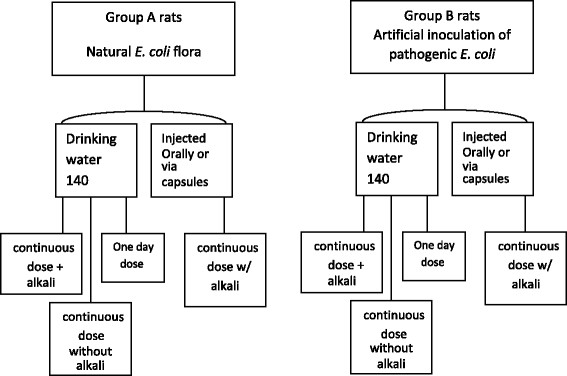
**The outline of methodology for the phage feeding biocontrol of intestinal** ***E. coli*** **in white rats.**

Two groups of albino rats, Rattus norvegicus, were subjected for phage biocontrol against *E. coli*. Group A, rats were fed with a phage cocktail of 140 phages without prior oral inoculation with human pathogenic *E. coli*. In this instance, the phage cocktail targets the naturally resident *E. coli* bacteria inside the animals’ gut. Group B, rats were fed for three weeks with phage cocktail of 140 phages along with, at the same time, oral inoculation of 60 human pathogenic E .coli isolates at concentration 10^8^ colony forming unit /ml (CFU/ml) in drinking water. Each inoculated *E. coli* isolate was recognized by three to four phage members of the used phage master mix bacteria [IPO-UK Patent Application No. 0822068]. As many animals act as reservoir for human pathogenic *E. coli*, group B provides an opportunity to test the biocontrol effect of the used phage cocktail against human *E. coli* pathogens that reside in the intestine of animals (Figure 1).

#### The control versus phage-biocontrolled fecal output of *E. coli*

The magnitude of *E. coli* phage control was measured in terms of log reduction (LR) of *E. coli* fecal output in tested rats. The total fecal output of *E. coli* in rats was estimated before and during the oral administration of the phage master mix in order to determine the pre-treatment level of *E. coli* as well as to determine the phage-based LR of *E. coli*. For unloaded rats with pathogenic *E. coli*, group A, the control fecal output of *E. coli* was measured in triplicate just before feeding the phages and was considered as the baseline from which changes of phage-driven *E. coli* output were measured in terms of LR in the same rats. For rats preloaded with pathogenic *E. coli*, group B, were sub-grouped into two sets. The first set, rats were not subjected to phage feeding which were used to measure, in triplicate, the control fecal output of the pathogenic plus resident *E. coli*. The second set, rats were subjected to phage feeding along with external *E. coli* inoculation. This set was used to measure, in triplicate, the phage-driven reduction of fecal output of *E. coli* in comparison with the first set.

#### Measurement of the *E. coli* fecal output

The fecal output of *E. coli* was measured by taking 0.2 g of feces diluted 1:5 w/v in PBS (Oxoid, UK). Afterwards, 10 ul of the net, 1:10, and 1:100 dilutions of the fecal solution were spotted on MacConckey agar and were incubated overnight at 43.4C. It is well known that *E. coli* bacteria are the only lactose fermenter enterobacteriacae that able to survive temperature of 43.4C. Next day, CFU were counted per each spot of inoculation. The concentration of fecal *E. coli*, in CFU/ml, was then calculated by incorporating the dilution and volume factors. Hence, *E. coli* bacteria were cultured selectively and then counted on daily basis.

#### Methods of the phage feeding

Phage feeding of rat animals was conducted via three methods. First, phage master mix was incorporated in drinking water at concentration of 10^7^ PFU/ml with/without 6% w/v Sodium bicarbonate and fed to animals via the rats’ water feeding bottle. In this way, rats took the phage cocktail orally every time they drink water. A group of rats was allowed to drink the phage-containing water for only one d while another group was allowed to drink the phage-containing water for 20 d in order to compare the phage-driven LR of fecal output of *E. coli* between the single day and continuous phage feedings. The second method of feeding of phage cocktail was achieved by injecting the phage master mix in lambda buffer at concentration of 10^7^ PFU/ml directly through oral route by using rats’ feeding bottles without further mixing with water. Four ml of phages suspension in lambda buffer with 6% w/v Sodium bicarbonate were managed to be injected orally to rats 3 times a day at 2 h intervals for 20 d. The third method of phage master mix feeding was done using absorbable vegetable capsules (ZeinPharma Germany GmbH, Germany) made of Hydroxypropyl Methylcellulose (HPMC) administered orally into the tested rats. Capsules were filled with 0.5 ml of phage cocktail in lambda buffer, 10^6^ PFU/ml of the phage master mix. Rats were given 3 capsules a day at 2 h intervals for 20 d (Figure 1).

#### Standardization of the phage master mix cocktail with animal feed

Different concentrations of phage master mix were evaluated to determine the optimal concentration that results in the best controlling effects of *E. coli* bacteria in rats’ intestine. The phage preparation was composed of a collection of 140 phages. Fifty ul from each bred phage were taken and collected in a 15 ml sterile tube (Vivantis, Italy). The phage cocktail was hence composed of 6 ml. According to the method of phage administration, the final concentration of phage feed was determined. In case of phage feeding in drinkable water, 1 ml of 10^6–13^ PFU/ml was added to 299 ml of tap water, i.e., 1:300 dilution. Therefore, the final concentration of phage-containing water was 10^3–10^ PFU/ml. The standardization procedures included administering phage cocktail via drinking water continuously for four days using different phage concentrations, 10^3–10^ PFU/ml. Then, the fecal output of *E. coli* was evaluated by standard bacteriological methods. It was shown that the increasing phage concentration gave increasing *E. coli* lowering. Nevertheless, 10^7^ PFU/ml led to LR of *E. coli* similar to that of 10^6^ PFU/ml and slightly better than higher concentrations in both phage drinking water and oral phage injection. Therefore, phage concentration of 10^7^ PFU/ml was considered optimal for phage feeding via drinking water and lambda buffer oral injection. On the other hand, for phage containing capsules, the standardization trials showed that the optimal concentration of phage master mix was one log lower than that used in drinking water or oral injection of lambda buffer, namely 10^6^ PFU/ml.

#### Phage shedding

Along with monitoring the effect of the phage feeding on the fecal output of *E. coli* in rat animals, the phage shedding from the animals’ intestine was evaluated throughout 3 weeks of continuous phage feeding via drinking water. The aim of this step was to assess the survival of the introduced phages inside rats’ intestine and assess the relationship between phage survival and its potential in lowering the intestinal *E. coli*. An EHEC-specific coliphage, CEH-183, was fed continuously with 6% w/v Sodium bicarbonate via both drinking water and oral phage injection thrice a day into rats that were previously fed with the target EHEC bacteria, EH-138, for successive 3 days in order to prepare the suitable host of the tested phage. On daily basis, 0.2 g of feces was diluted 1:5 w/v in PBS. Ten ul of the net, 1:10, and 1:100 dilutions of the fecal solution were then spotted on an 18 h bacterial lawn of EH-183. Next day, the resulted plaques were enumerated in each spot; the concentration of shed phages was then calculated in terms of PFU/ml. Therefore, the level of intestinal phage shedding was evaluated with time to probe the effect of the immunity of the tested animals on administered phages. Moreover, the level of phage shedding was essential in giving the optimal time for the peak phage replication and availability inside animals’ intestine and testing whether this timing is coordinated with the optimal time of *E. coli* lowering by the effect of fed phages or not. This was important to determine the survival of phages and the effect of the animals’ immunity on the phages survival. This would help determine the exact timing of the maximal phage biocontrol.

### Statistical analysis

The data was analyzed and graphics were produced using SPSS software version 12.0.0.1 as well as MS EXCEL software version 2000. Logarithmic values in terms of log_10_ CFU/ml and log_10_ PFU/ml for bacterial and phage concentrations respectively were used. The logarithmic mean, mean log_10_ CFU/ml or mean log_10_ PFU/ml, were calculated by averaging the individual log_10_ CFU/ml or log_10_ PFU/ml values [20]. The mean log reduction (LR) in CFU/ml was calculated by subtracting the mean log_10_ CFU/ml of negative control from mean log_10_ CFU/ml of test samples. Mean LR CFU/mL ≥ 1 was considered as significant. The standard error (SE) of mean LR was calculated according to the following equation [20]:

(1) $$\mathrm{SE}\;=\;\left[\left(\mathrm{Variance}\;\mathrm{mean}\;\log_{10}\mathrm{CFU}/\mathrm{mL}\;\mathrm{control}\;÷\;N\right)\;+\;\left(\mathrm{Variance}\;\mathrm{mean}\;\log_{10}\mathrm{CFU}/\mathrm{mL}\;\mathrm{test}\;÷\;N\right)\right]\;\mathrm{sq}.\;\mathrm{root}$$

Where N equals to sample size

## Results and discussion

### Standardization of the administered phages

It was found that the optimal concentration of applied phage master mix was 10^7^ PFU/ml. This concentration gave results pretty similar to that of 10^6^ PFU/ml and better than higher concentrations (Table 1). On the other hand, although the phage cocktail in drinking water was refreshed daily, the phage survival in tap water was measured. It was found that significant phage decline (>1 log) started only after 6 d of the preparation of phage-containing drinking water. This decline in phage concentration was most likely due to phage decay. Hence, changing the phage-containing drinking water should be done every 6 days in maximum. For the sodium bicarbonate added to drinking water, it was found that 6% concentration w/v was optimal as higher concentrations affected negatively the life span of phage cocktail while lower concentrations have lower anti-stomach acid neutralization [data not shown].

**Table 1:** **Mean LR (log**_**10**_**CFU/ml) of fecal output of** ***E. coli*** **in rat animals treated with different concentrations of phage master mix in drinking water**

**Phage concentration PFU/ml in drinking water**	**Mean LR (log**_**10**_**CFU/ml)**	
	**2 days**	**4 days**
1x10^3^	0 ± 0.0	1.2 ± 0.08
1x10^4^	0.1 ± 0.02	1.2 ± 0.1
1x10^5^	0.3 ± 0.06	1.41 ± 0.17
1x10^6^	0.28 ± 0.07	1.36 ± 0.07
1x10^7^*(optimal)*	0.6 ± 0.04	1.8 ± 0.14
1x10^8^	0.6 ± 0.08	1.73 ± 0.2
1x10^9^	0.5 ± 0.04	1.7 ± 0.15
1x10^10^	0.4 ± 0.09	1.5 ± 0.2

### Phage biocontrol

#### Group A (rats with normal intestinal *E. coli* flora)

##### Phage feeding via drinking water

Single day phage feeding in drinking water:

The *E. coli* fecal output started to decline after day 3 from the beginning of phage feeding; the peak of LR of *E. coli* was at days four to six. The maximal phage-based LR of *E. coli* fecal output was 1.225 log units, which is a significant reduction. From day seven, the phage-driven LR of *E. coli* output was started to vanish and *E. coli* count started to rise again reaching 2.sx10^5^ CFU/ml at day 11 which was close to the negative control concentration of the *E. coli* output (Table 2).

**Table 2:** **LR of** ***E. coli*** **fecal output due to the effect of single day feeding of phage master mix**

**Phage feed at 1×10**^**7**^**PFU/ml via drinking water for one d**	***E. coli*** **CFU/ml**	**Mean LR (log**_**10**_**CFU/ml)**
Time zero (control load)	1.8 x 10^5^	-
Day 1	7 x 10^4^	0.4 ± 0.02
Day 2	6.3 x 10^4^	0.45 ± 0.08
Day 3	6.9 x 10^4^	0.39 ± 0.07
Day 4	1.3 x 10^4^	1.11 ± 0.12
Day 5	1 x 10^4^	1.22 ± 0.18
Day 6	1.5 x 10^4^	1.04 ± 0.22
Day 7	4.7 x 10^4^	0.55 ± 0.1
Day 8	1 x 10^5^	0.22 ± 0.08
Day 9	2 x 10^5^	−0.07 ± 0.01
Day 10	2.5 x 10^5^	−0.17 ± 0.06
Day 11	2.2 x 10^5^	−0.1 ± 0.04

##### Continuous phage feeding in drinking water:

The results provided evidence that intestinal *E. coli* started to decline after 3 days from the beginning of phage feeding, the peak of *E. coli* decline was day 5 to day 7 after the beginning of phage feeding. Lowest fecal output of *E. coli* was 10^2^ CFU/ml while the control load, time zero, was 2×10^4^ CFU/ml. The *E. coli* phage-driven LR was maximally 2.3 log units at day 7, which is a highly significant decline. From day 14, the phage-driven LR of *E. coli* output was started to vanish and *E. coli* count started to rise again reaching 1x10^4^ CFU/ml at day 20 which was close to the control load of the *E. coli* output (Table 3).

**Table 3:** **LR of** ***E. coli*** **fecal output due to the effect of continuous feeding of phage master mix for 20 d via drinking water**

**Phage feed at 1×10**^**7**^**PFU/ml via drinking water for 20 d**	***E. coli*** **CFU/mL**	**Mean LR (log**_**10**_**CFU/ml)**
Time zero (control load)	2 x 10^4^	-
Day 1	2 x 10^4^	0 ± 0.0
Day 2	2.4 x 10^4^	−0.08 ± 0.007
Day 3	1 x 10^4^	0.3 ± 0.01
Day 4	2 x 10^3^	1 ± 0.08
Day 5	2 x 10^2^	2 ± 0.12
Day 6	2 x 10^2^	2 ± 0.15
Day 7	1 x 10^2^	2.3 ± 0.24
Day 8	4 x 10^2^	1.7 ± 0.2
Day 10	6 x 10^2^	1.52 ± 0.18
Day 12	1 x 10^3^	1.3 ± 0.1
Day 14	4.8 x 10^3^	0.61 ± 0.06
Day 16	1 x 10^3^	1.3 ± 0.09
Day 20	1 x 10^4^	0.3 ± 0.04

##### Continuous phage feeding via drinking water with alkali:

*E. coli* started to decline after day 2 from the beginning of phage feeding. The lowest fecal output of *E. coli* was 10^2^ CFU/ml at 7^th^ day. The maximal phage-driven LR of *E. coli* was 2.78 log units, which is a significant decline. From day 14, the phage-driven decline of *E. coli* output was started to vanish and *E. coli* count started to rise again reaching 3×10^4^ CFU/ml at day 20 which was close to the control load of the *E. coli* fecal output (Table 4).

**Table 4:** **LR of** ***E. coli*** **fecal output due to the effect of continuous feeding of phage master mix for 20 d via drinking water with alkali**

**Phage feed at 1×10**^**7**^**PFU/ml via drinking water for 20 d**	***E. coli*** **CFU/mL**	**Mean LR (log**_**10**_**CFU/ml)**
Time zero (control)	6 x 10^4^	-
Day 1	4 x 10^4^	0.17 ± 0.02
Day 2	2 x 10^4^	0.47 ± 0.03
Day 3	2 x 10^4^	0.47 ± 0.02
Day 4	1 x 10^3^	1.78 ± 0.18
Day 5	1 x 10^3^	1.78 ± 0.09
Day 6	2 x 10^3^	1.47 ± 0.1
Day 7	1 x 10^2^	2.78 ± 0.14
Day 8	2 x 10^2^	2.47 ± 0.22
Day 10	1 x 10^3^	1.78 ± 0.12
Day 12	1 x 10^3^	1.78 ± 0.15
Day 14	1 x 10^4^	0.78 ± 0.08
Day 16	2 x 10^3^	1.47 ± 0.11
Day 20	3 x 10^4^	0.3 ± 0.03

#### Phage feeding via oral injection

In this method of administration, one protocol was pursued which is continuous feeding of phages for 20 d with alkali because it proved to be the best protocol in the phage feeding via water drinking. Rat animals were injected orally with phage suspension three times a day. It was found that the baseline *E. coli* output prior to phage feeding was 2.3×10^5^ CFU/ml, noticeable decline started after day 2 and the lowest *E. coli* count was found in days 6 to 10, peak of decline was at 6^th^ day, 4×10^3^ CFU/ml when the maximal phage-driven LR of intestinal *E. coli* was 1.76 log units. At day 12, *E. coli* output started to rise again till reaching 1.2×10^5^ CFU/ml at day 20 (Table 5).

**Table 5:** **LR of** ***E. coli*** **fecal output due to the effect of continuous feeding of phage master mix for 20 d via oral injection with alkali**

**Phage feed at 1×10**^**7**^**PFU/ml via oral injection of phages for 20 d**	***E. coli*** **CFU/mL**	**Mean LR (log**_**10**_**CFU/ml)**
Time zero (control)	2.3 x 10^5^	-
Day 1	1.6x 10^5^	0.15 ± 0.03
Day 2	9 x 10^4^	0.4 ± 0.02
Day 3	1 x 10^5^	0.36 ± 0.07
Day 4	1 x 10^4^	1.36 ± 0.15
Day 5	1 x 10^5^	0.36 ± 0.09
Day 6	4 x 10^3^	1.76 ± 0.18
Day 7	7 x 10^3^	1.51 ± 0.22
Day 8	9 x 10^3^	1.4 ± 0.13
Day 10	8 x 10^3^	1.45 ± 0.1
Day 12	1 x 10^5^	0.36 ± 0.05
Day 14	5 x 10^4^	0.66 ± 0.03
Day 16	5 x 10^4^	0.66 ± 0.06
Day 20	1.2 x 10^5^	0.28 ± 0.04

#### Phage feeding via oral capsules

In this method of administration, one protocol was pursued which is the continuous feeding. It was found that the baseline E .coli output prior to phage feeding was 1.8×10^5^ CFU/ml. The decline of *E. coli* count was maximal in days 3 to 10; peak of decline was at 6^th^ day, 1.7×10^2^ CFU/ml when the phage-driven LR of intestinal *E. coli* was 3.02 log units. At day 12, *E. coli* count started to rise again reaching 2.3 × 10^4^ CFU/ml. (Table 6).

**Table 6:** **LR of** ***E. coli*** **fecal output due to the effect of continuous feeding of phage master mix for 20 d via oral administration of phage-containing capsules**

**Phage feed at 1×10**^**6**^**PFU/ml via phage-containing capsules for 20 d**	***E. coli*** **CFU/mL**	**Mean LR (log**_**10**_**CFU/ml)**
Time zero (control)	1.8x10^5^	-
Day 1	1.1x 10^5^	0.21 ± 0.04
Day 2	9 x 10^3^	1.3 ± 0.15
Day 3	1 x 10^3^	2.25 ± 0.07
Day 4	8 x 10^3^	1.35 ± 0.2
Day 5	7.6 x 10^2^	2.37 ± 0.16
Day 6	1.7 x 10^2^	3.02 ± 0.22
Day 7	2.8 x 10^2^	2.8 ± 0.18
Day 8	1 x 10^3^	2.25 ± 0.24
Day 10	8 .5x 10^3^	1.32 ± 0.1
Day 12	2.3 x 10^4^	0.89 ± 0.07
Day 14	1 x 10^4^	1.25 ± 0.1
Day 16	1 x 10^5^	0.25 ± 0.05
Day 20	2x10^5^	−0.04 ± 0.006

#### Group B (rats preloaded with human pathogenic *E. coli*)

This group of rat animals was fed continuously for 20 d with heavily contaminated 60 human pathogenic isolates. At the same time, 140 of corresponding lytic bred phages were given via drinking water, oral injection, and phage-containing capsules continuously for 20 d.

#### Baseline fecal output of *E. coli* in group B rats before phage feeding (pretreatment)

There was a need to monitor the baseline level of *E. coli* output in group B rats that were continuously administered a contaminated, 10^8^ CFU/mL, drinking water orally without phage cocktail feed. After loading up rat intestines with human pathogenic *E. coli*, it was found that *E. coli* output at day 0 was 2.1 × 10^5^ CFU/ml and *E. coli* count started to increase after 24 h, peak of increase was at day 6, 1.1 × 10^7^ CFU/ml or 1.71 log units. Then, fecal output of *E. coli* started to decline reaching 3.4 × 10^5^ CFU/ml at day 20 (Table 7).

**Table 7:** **Fecal output of** ***E. coli*** **increased with the effect of continuous human pathogenic** ***E. coli*** **feeding for 20 d via drinking water**

**Pathogenic** ***E. coli*** **feed at 1×10**^**8**^**CFU/ml with water drink for 20 d**	**Total** ***E. coli*** **output count (CFU/ml)**
Time zero	2.1 x 10^5^
Day 1	2.4x 10^5^
Day 2	3.2x 10^6^
Day 3	7 x 10^6^
Day 4	6.3 x 10^6^
Day 5	8.5 x 10^6^
Day 6	1.1 x 10^7^
Day 7	1 x 10^7^
Day 8	9 x 10^6^
Day 10	8.7x 10^5^
Day 12	7 x 10^5^
Day 14	5.3 x 10^5^
Day 16	5.1 x 10^4^
Day 20	3.4 x 10^5^

#### Phage feeding via drinking water

Rats that were already fed with heavily contaminated 60 *E. coli* pathogens, were fed with the phage master mix in separate feeding bottle simultaneously for 20 d. Since the protocol of phage feeding with alkali for 20 d was shown as the best in group A rats, it was the only drinking protocol pursued in group B rats. It was intended to monitor is there any decline of the total *E. coli* output when the pathogenic *E. coli*-inoculated rats were fed with the specific phages of the inoculated pathogens. And whether phage feeding is able to stop or reverse the *E. coli* output upsurge. It was found that phage feeding succeeded in reversing the upsurge of total *E. coli* output starting from day 2 and severe lowering of *E. coli* output was peaked in days 5 to 8; peak lowering was in day 7, 8.2×10^2^ CFU/ml, when the *E. coli* level in the pre-treatment rats was 1×10^7^ CFU/ml. Therefore, the expected phage-driven LR of *E. coli* output was the difference between the *E. coli* output in group B rats without phage feeding, 1×10^7^ CFU/ml, and that seen in group B rats with phage feeding, 8.2×10^2^ CFU/m, namely 4.07 log units. After day 10, the *E. coli* output started to rise again reaching 4.6×10^5^ CFU/ml (Table 8).

**Table 8:** ***E. coli*** **output decline in group B rats due to the continuous phage feeding for 20 d via water drinking with alkali**

**Phage feed at 1×10**^**7**^**PFU/ml via drinking water for 20 d**	**Total** ***E. coli*** **output count (CFU/mL)**	**Mean LR (log**_**10**_**CFU/ml)**
Time zero	1.4 x 10^5^	-
Day 1	1.9x 10^5^	0.1 ± 0.03
Day 2	3.5x 10^5^	0.96 ± 0.09
Day 3	4.1x 10^5^	1.23 ± 0.13
Day 4	7.4 x 10^4^	1.93 ± 0.11
Day 5	6.8 x 10^3^	3.09 ± 0.28
Day 6	5.2 x 10^3^	3.32 ± 0.24
Day 7	8.2 x 10^2^	4.08 ± 0.3
Day 8	8.7 x 10^2^	4.01 ± 0.23
Day 10	4x 10^3^	2.33 ± 0.16
Day 12	6.3 x 10^3^	2.04 ± 0.14
Day 14	3 x 10^4^	1.24 ± 0.1
Day 16	5.7 x 10^5^	−1.04 ± 0.06
Day 20	3 x 10^5^	0.05 ± 0.002

#### Phage feeding via oral injection

Phage feeding was conducted by oral injections of lambda buffer solution with 6% w/v Sodium bicarbonate (alkali) thrice a day for 20 d. It was found that results were relatively similar but a bit less prominent than that of water drinking protocol done for group B rats. The lowering in *E. coli* output was started in day 2; peaked in days 5–8. The maximal LR was at day 7, 3.45 log units (Table 9).

**Table 9:** ***E. coli*** **output decline due to the continuous phage feeding for 20 d via oral injection of group B rats**

**Phage feed at 1×10**^**7**^**PFU/ml via oral injection for 20**	**Total** ***E. coli*** **output count (CFU/mL)**	**Mean LR (log**_**10**_**CFU/ml)**
Time zero (control –ve)	2.4 x 10^6^	-
Day 1	2.3x 10^5^	0.01 ± 0.003
Day 2	2.5x 10^5^	1.1 ± 0.08
Day 3	3.2x 10^5^	1.33 ± 0.12
Day 4	7.3 x 10^4^	1.9 ± 0.14
Day 5	4.2 x 10^4^	2.3 ± 0.09
Day 6	8.4 x 10^3^	3.1 ± 0.24
Day 7	3.5 x 10^3^	3.45 ± 0.2
Day 8	8.1 x 10^3^	3.04 ± 0.3
Day 10	5.8x 10^3^	2.17 ± 0.17
Day 12	7.1 x 10^4^	0.99 ± 0.1
Day 14	2 x 10^5^	0.42 ± 0.07
Day 16	7 x 10^4^	−0.13 ± 0.02
Day 20	3 x 10^5^	0.054 ± 0.006

#### Phage feeding via oral capsules

Phage feeding was conducted by administering phage-containing capsules thrice a day for 20 days. It was found that the lowering in *E. coli* output was started in day 2; peaked in days 5–8. The maximal lowering was in day 6, 2.6 ×10^2^ CFU/ml and the peak LR was 4.62 log units. *E. coli* output then started to increase after day 10 reaching 5.3×10^5^ CFU/ml at day 20 (Table 10).

**Table 10:** ***E. coli*** **output decline due to the continuous phage feeding for 20 d via phage-containing capsules**

**Phage feed at 1×10**^**6**^**PFU/ml in vegetable capsules for 20 d**	**Total** ***E. coli*** **output count (CFU/ml)**	**Mean LR (log**_**10**_**CFU/ml)**
Time zero (control –ve)	3.1 x 10^6^	-
Day 1	3.7x 10^6^	−1.18 ± 0.07
Day 2	6.2x 10^5^	0.71 ± 0.05
Day 3	9x 10^4^	1.89 ± 0.13
Day 4	2.8 x 10^4^	2.35 ± 0.21
Day 5	3.2 x 10^3^	3.42 ± 0.026
Day 6	2.6 x10^2^	4.62 ± 0.32
Day 7	3.7 x 10^2^	4.43 ± 0.028
Day 8	6.9 x 10^2^	4.1 ± 0.17
Day 10	7.3x 10^3^	2.07 ± 0.09
Day 12	4.8 x 10^4^	1.16 ± 0.14
Day 14	2.3 x 10^5^	0.36 ± 0.05
Day 16	4.4 x 10^5^	−0.93 ± 0.1
Day 20	5.3x10^5^	−0.19 ± 0.04

### Phage shedding during phage feeding

It was found that phage shedding increased gradually after day 1 and peaked in days 4–12 then it declined again which is the reverse pattern seen for *E. coli* output. There was no remarkable difference between the pattern of phage shedding between oral injection and phage-containing water drink (Table 11).

**Table 11:** Phage shedding from the studied rats during phage feeding biocontrol

**Phage feed at 1×10**^**7**^**PFU/ml with water drink and oral injection for 20 d**	**Total phage shedding (PFU/ml) via water drink**	**Total phage shedding (PFU/ml) via oral injection**
Time zero (control –ve)	0	0
Day 1	0	40
Day 2	1.18 x 10^3^	8.0 x 10^2^
Day 3	1.22 x 10^3^	9.4 x 10^2^
Day 4	2.6 x 10^4^	1.2 x 10^3^
Day 5	8.3 x 10^4^	5.2 x 10^3^
Day 6	2.7 x 10^5^	3.8 x 10^4^
Day 7	5.4 x 10^5^	4.1 x 10^5^
Day 8	1.5 x 10^4^	7.9 x 10^4^
Day 10	3.7 x 10^3^	3.8 x 10^3^
Day 12	7.2 x 10^2^	8.7 x 10^2^
Day 14	1.1 x 10^2^	64
Day 16	8.9	6
Day 20	0	0

Escherichia coli O157 and other EHEC are believed to be carried asymptomatically by cattle, cows and sheep [5]. Shedding of the organism in the faeces of animals is intermittent and can be exacerbated by a variety of factors [21] including stress and feed types. Therefore, a thorough approach is needed to control the microbial load of the pathogenic *E. coli* where bovine and other warm-blooded animal’s feacal contamination is possible. The ability of phages to recognize precisely their hosts rendered the use of phages as antibacterial agents favorable because of the fact that broad-spectrum antibiotics kill the target bacteria along with all beneficial bacteria in the farm or in the organism body [10].

Unfortunately this was the bright side of the story; on the other hand, phage specificity imposed a complicated dilemma, which is the difficulty of finding sufficient phage strains which could cover all or most of bacterial host strains. Thus, the inability to cover all strains of certain bacterial species along with the easy development of evolutionary resistance by the bacteria against their phages, have made phage therapy or biocontrol is unsuccessful [15]. This was worked out by the novel techniques of phage breeding and optimization pursued by our team [IPO-UK Patent Application No. 0822068]. This prepared the suitable background for formulating highly lytic and optimized cocktail of large number of anti- *E. coli* phages that are able to apply predator effect on hundreds of target bacteria. For this reason, phage cocktail of anti- *E. coli* bacteria, the phage master mix, was used to control and predate target strains of *E. coli* whether reside naturally inside rat animals or artificially inoculated human pathogenic *E. coli* in rat guts. The aim of this was to evaluate the capability of phage cocktail to suppress the microbial load of *E. coli* represented by the reduction in *E. coli* fecal output. This has special importance because most cases of transmission of pathogenic *E. coli* including O157:H7 are related to fecal contamination by animals to the food chain [22]. Therefore, it was conceived that decreasing or abolishing the fecal contamination would decrease largely the chances for human beings to contract *E. coli* infection.

Accordingly, in the current study, it was found that the used phage cocktail was remarkably successful in lowering the *E. coli* output of both *E. coli* flora and externally inoculated pathogenic *E. coli* bacteria in significant manner. Three protocols were used of phage feeding via drinking, phage feeding for one day, for 20 d, and 20 d with alkali. It was found that single day phage feed was effective but much less efficient than continuous phage feed for 20 d with or without alkali. In single day feed, brief *E. coli* decline for 3 days, 4^th^ to 6^th^ day and maximal LR was 1.22 log units and then at 7^th^ day *E. coli* output began to rise again due to the loss of the phage lysing effect. In 20 d phage feed, the decline of *E. coli* load began after 3 days with extended lowering phase ranged from 5^th^ days to 10^th^ day and the maximal LR was 2.3 log units without alkali and 2.78 with alkali. The difference in LR of *E. coli* between single day and 20 d continuous feed was remarkable, 1.56 log units. This indicated that there was a remarkable enhancement in phage-driven *E. coli* control by using daily phage feed rather than one or few days feeding. Moreover, there was another little enhancement when alkali was used which could counteract the acidity of rats’ stomach.

On the other hand, using continuous phage feed by oral injection of phages-containing lambda buffer with alkali did not give better results as was expected. The oral injection of phages resulted in 1.02 log units less LR than that of continuous phage feed via drinking water. This might be attributed to the frequency of the oral injections of phages into rats per day. Rats were orally injected thrice a day and this most probably was less effective than the available phage-containing drinking water given that rats drink water, roughly, every 10–15 min which guarantees undisrupted supply of the phage cocktail. However, it is expected that more frequent oral injections of phages per day might lead to much better results. However, the current study provided evidence that oral injection of phages is not a practicable option as injecting phages orally needs much efforts and equipment. On the contrary, feeding phages, at 10^6^ PFU/ml, via vegetable capsules proved to be superior. Phage containing capsules administered 3 times a day succeeded to achieve LR > 3 log units, 3.02, while other routes of demonstration did not. This might be attributed to the better protection of phages from stomach acidity than using alkali.

For group B rats, human pathogenic *E. coli* were fed to rat animals in parallel with administering specific coliphage cocktail in order to measure the potential of the phage master mix in controlling the externally inoculated pathogenic *E. coli* as well as the resident *E. coli* flora of rats. Rats fed with heavy dose of human pathogenic *E. coli* tolerated well these bacteria in that a remarkable increase was seen in the total *E. coli* output after feeding 10^8^ CFU/ml of *E. coli* daily for 20 d. After just 6 d of the inoculation of the pathogenic *E. coli*, the fecal output of *E. coli* was 1.8 log units higher than before; *E. coli* output increased after 4 to 10 d and this upsurge of *E. coli* was then started to decline progressively. Similar to the behavior of the introduced phages, it is prudent to think that the mucosal immunity of rats’ gut was responsible for this lowering of *E. coli* upsurge. However, our hypothesis is that if rat animals, which were already loaded with external *E. coli*, were challenged with phage feeding specific to the same collection of external *E. coli,* then *E. coli* output upsurge should start to decline before the immunity develops, before 10^th^ day, just like the case for the group A rats when the natural *E. coli* flora was challenged by phage feeding. A striking *E. coli* output lowering was achieved by phage feed for 20 d with alkali via water drink, oral injection of phage containing lambda buffer, and phage containing capsules. The fecal output of *E. coli* did not increase in rats received phages concomitantly with pathogenic *E. coli*. Moreover, there was huge lowering of *E. coli* output mainly after five to eight d from phage-bacteria feeding. The peak LR of fecal output of *E. coli* was 4.08, 3.45, and 4.62 log units in rats subjected to 20 d phage feed via drinking water, oral injection, and capsules respectively. Hence, phage feed reversed the upsurge of *E. coli* output. This provided evidence that phage feed biocontrol is more efficient in combating the pathogenic non natural *E. coli* flora than combating natural flora bacteria. Therefore, phage feed proved to be useful in suppressing both animals *E. coli* flora and more efficiently the external *E. coli* pathogens in their intestines.

For more confidence in the phage effect in controlling the residing E .coli inside animals’ intestine, phage shedding was explored, it was found that phage shedding peaks with the peak decline of *E. coli* output and then phage shedding starts to abolish reaching zero at the end of the third week. There was no significant difference between phage shedding in animals fed with phages via water drink or via oral injection. The pattern seen in this study for phage shedding and phage-driven *E. coli* output control was believed to be immune response -dependent. Rats developed effective immunity against all introduced phages as well as introduced human pathogenic *E. coli* isolates. The developed immune reaction was thought first as *E. coli* resistance against invading phages but in vitro plaque assay of *E. coli* bacteria isolated from fecal output of rats before and after phage feeding showed that *E. coli* bacteria after phage feeding were as sensitive to the fed phages as *E. coli* bacteria isoalted before phage feeding [data not shown]. Therefore, the gradual loss of the phage-driven *E. coli* control was most likely attributed to immunity development. Moreover, phage shedding pattern confirmed this explanation as introduced phages were eliminated gradually at the same period of introduced bacteria elimination. In addition, it is noteworthy to mention that some rat animals that were used in phage feeding two month earlier were rechallenged again with the same phage cocktail. The phage biocontrol was null even two months passed since last time phage feeding was done [data not shown]. This finding was another proof for the immunity hypothesis for the termination of the phage feeding effect control.

In recent years, there has been an obsessive question, whether phages are safe for use in biocontrol or therapy. No signs or symptoms were found when phage cocktail was given. It was cautioned that high number of phages introduced into rat guts might lead to extensive abolishment of *E. coli* flora; but, fortunately, it was found that *E. coli* load was lowered but not abolished completely in a way that no signs and symptoms of disturbance in bowel habit was noticed nor any other change of animals behavior was observed. This was a clue on the harmless use of phages for lowering *E. coli* load in animals. Given that such phage-based biocontrol is conducted only six to eight days before animal slaughter, say cattle, hence, long-term adverse effects of using phages might be zero. The safety of phages was assured by Duckworth and Gulig [23] who stated that there has been no evidence that exposure to phage particles, even ones normally associated with disease-causing bacteria, can actually result in the occurrence of human disease. In addition, the recent FDA approval of Listeria-specific bacteriophage preparations for food preservation has opened the door to new applications of these natural bacterial killers taken that bacteriophages are viruses that only infect and lyse bacterial cells and are harmless to mammalians [24]. Another safety aspect might be taken into consideration, phages replicate at the site of infection or wherever the host bacteria are present while phages are absent in sterile areas ensuring an optimal self-adjusting dose of phages which is not found in other modes of non-biological antimicrobial agents [25].

## Conclusions

Taken together, it was concluded that phage feed as a mean of biocontrol for lowering the microbial load of *E. coli* bacteria inside animals’ intestine proved to be successful. The suppression of *E. coli* output reached 3 log units for rats intestine resident *E. coli* and 4.62 log units for both externally inoculated and flora *E. coli*. And phage feeding using phage cocktail composed of high number, 140, of specific bred phages coliphages ensured the wide coverage of *E. coli* strains and substrains leading to efficient *E. coli* load suppression. Moreover, the best administration route of phage cocktail was found to be via vegetables capsules followed by drinking water which were more efficient that injecting phages directly into rat throat. In addition, phage cocktail fed continuously for 20 d was the best protocol of feeding and better to be accompanied by a small percentage of biologically safe alkali such as Sodium bicarbonate that resulted in better phage control. The pattern of the phage-based *E. coli* biocontrol highlighted that timing of the preharvest animal biocontrol is the most critical step deciding the success of the phage biocontrol. It was concluded that the 6^th^, 7^th^, and 8^th^ days after the beginning of the phage feeding were the best days to slaughter the animal when the highest phage shedding and the lowest *E. coli* output were seen. Even though 6^th^ or 7^th^ day might be better than 8^th^ day, it is preferred to do slaughtering in the 8^th^ day to allow 2 days of minimal *E. coli* output before slaughtering. This can ensure that *E. coli* contamination into the food chain will be greatly minimized.

## Competing interests

Authors confirm that there is no conflict of interests of any kind.

## Authors’ contributions

AS designed and conducted the research, SAAJ and F designed the research. All authors read and approved the final manuscript.

## References

[B1] Kaper JB Nataro JP Mobley HLT Pathogenic *Escherichia coli* Nat Rev Microbiol 2004 2 123 140 10.1038/nrmicro818 15040260

[B2] Carone BR Xu T Murphy KC Marinus MG High incidence of multiple antibiotic resistant cells in cultures of in enterohemorrhagic Escherichia coli O157:H7 Mutat Res 2014 759 1 8 10.1016/j.mrfmmm.2013.11.008 PMC3913999 24361397

[B3] Wall PG McDonnell RJ Adak GK Cheasty T Smith HR Rowe B General outbreaks of vero cytotoxin producing Escherichia coli O157 in England and Wales from 1992 to 1994 Commun Dis Rep CDR Rev 1996 6 26 33 8777442

[B4] Hawker J Begg NB Blair B Reintjes R Weinberg J Communicable Disease Control Handbook 2001 Blackwell, Oxford

[B5] Rasmussen MA Casey TA Environmental and food safety aspects of Escherichia coli O157:H7 infections in cattle Crit Rev Microbiol 2001 27 57 73 10.1080/20014091096701 11450854

[B6] Hussein HS Prevalence and pathogenicity of Shiga toxin-producing Escherichia coli in beef cattle and their products J Anim Sci 2007 85 E63 E72 10.2527/jas.2006-421 17060419

[B7] Yoder JS Blackburn BG Craun GF Hill V Levy DA Chen N Lee SH Calderon RL Beach MJ Surveillance for waterborne-disease outbreaks associated with recreational water-United States MMWR Surveill Summ 2004 53 1 22 15499306

[B8] Grandgirard D Loeffler JM Fischetti VA Leib SL Phage lytic enzyme Cpl-1 for antibacterial therapy in experimental pneumococcal meningitis J Infect Dis 2008 197 1519 1522 10.1086/587942 18471063

[B9] Rea MC Alemayehu D Ross RP Hill C Gut solutions to a gut problem: bacteriocins, probiotics and bacteriophage for control of Clostridium difficile infection J Med Microbiol 2013 62 Pt 9 1369 1378 10.1099/jmm.0.058933-0 23699066

[B10] Merril CR Scholl D Adhya SL The prospect for Bacteriophage therapy in Western medicine Nat Rev Drug Discov 2003 2 489 497 10.1038/nrd1111 12776223

[B11] Wittebole X De Roock S Opal SM A historical overview of bacteriophage therapy as an alternative to antibiotics for the treatment of bacterial pathogens Virulence 2014 5 1 226 235 10.4161/viru.25991 23973944 PMC3916379

[B12] Barrow PA Soothill JS Bacteriophage therapy and prophylaxis: rediscovery and renewed assessment of potential Trends Genet 1997 5 268 271 10.1016/S0966-842X(97)01054-8 9234508

[B13] Carlton RM Phage therapy: past history and future prospects Arch Immunol Ther Exp 1999 47 267 274 10604231

[B14] Verbeken G Pirnay JP De Vos D Jennes S Zizi M Lavigne R Casteels M Huys I Optimizing the European regulatory framework for sustainable bacteriophage therapy in human medicine Arch Immunol Ther Exp (Warsz) 2012 60 3 161 172 10.1007/s00005-012-0175-0 22527355

[B15] Vieu JF Fabre J Les Bacteriophages Fraite de Therapeutique 1975 Vol. Serums et Vaccins. Flammarion, Paris 337 400

[B16] Smith HW Huggins MB Effectiveness of phages in treatingexperimental Escherichia coli diarrhoea in calves, piglets and lambs J Gen Microbiol 1983 129 2659 2675 6355391 10.1099/00221287-129-8-2659

[B17] Smith HW Huggins MB Shaw KM The control of experimental Escherichia coli diarrhoea in calves by means of bacteriophages J Gen Microbiol 1987 133 1111 1126 3309177 10.1099/00221287-133-5-1111

[B18] Tauxe RV Emerging foodborne diseases: an evolving public health challenge Emerg Infect Dis 1997 3 425 434 10.3201/eid0304.970403 9366593 PMC2640074

[B19] Topley WWC Wilson GS Principles of bacteriology, virology and immunity 1990 B.C. Decker Publisher, London, United Kingdom

[B20] Drake D Doyle RJ Assessment of antimicrobial activity against biofilms Microbial growth in biofilms vol. 2 2001 Academic press, London, UK 373 375

[B21] Garber L Wells S Shroeder-tucker L Factors associated with the faecal shedding of verotoxin-producing Escherichia coli O157 on dairy farms J Food Prot 1999 62 307 312 10419200 10.4315/0362-028x-62.4.307

[B22] Callaway TR Anderson RC Edrington TS Genovese KJ Bischoff KM Poole TL Jung YS Harvey RB Nisbet DJ What are we doing about Escherichia coli O157:H7 in cattle? J Anim Sci 2004 82 E-Suppl E93 E99 15471819 10.2527/2004.8213_supplE93x

[B23] Duckworth DH Gulig PA Bacteriophages: potential treatment for bacterial infections BioDrugs 2002 16 57 62 10.2165/00063030-200216010-00006 11909002

[B24] Chibeu A Agius L Gao A Sabour PM Kropinski AM Balamurugan S Efficacy of bacteriophage LISTEXP100 combined with chemical antimicrobials in reducing Listeria monocytogenes in cooked turkey and roast beef Int J Food Microbiol 2013 167 2 208 214 10.1016/j.ijfoodmicro.2013.08.018 24125778

[B25] Drilling A Morales S Boase S Jervis-Bardy J James C Jardeleza C Tan NC Cleland E Speck P Vreugde S Wormald PJ Safety and efficacy of topical bacteriophage and ethylenediaminetetraacetic acid treatment of Staphylococcus aureus infection in a sheep model of sinusitis Int Forum Allergy Rhinol 2014 4 3 176 186 10.1002/alr.21270 24449635

